# Factors associated with condom use among HIV-positive women living in Atlanta, Georgia

**DOI:** 10.1371/journal.pone.0225406

**Published:** 2019-12-13

**Authors:** Priya R. Gursahaney, Sarah Cordes, Ighovwerha Ofotokun, Kristin M. Wall, Denise J. Jamieson, Lisa B. Haddad

**Affiliations:** 1 University of Cincinnati College of Medicine, Department of Obstetrics and Gynecology, Cincinnati, Ohio, United States of America; 2 Emory University School of Medicine, Department of Gynecology and Obstetrics, Atlanta, Georgia, United States of America; 3 Emory University School of Medicine, Department of Medicine, Infectious Disease Division and Grady Health Care System, Atlanta, Georgia, United States of America; 4 Emory University Rollins School of Public Health, Department of Epidemiology, Atlanta, Georgia, United States of America; David Geffen School of Medicine at UCLA, UNITED STATES

## Abstract

**Objectives:**

Consistent condom use is essential to reducing heterosexual transmission of HIV. African Americans are disproportionately affected by HIV in the United States despite comprising a small percentage of the population. Our objectives were to evaluate factors associated with self-reported condom use in a cohort of predominantly African American women receiving HIV care in Atlanta, Georgia.

**Methods:**

A cross-sectional study of reproductive knowledge, attitudes, and practices among adult, sexually-active, HIV-positive women attending the Grady Infectious Disease Clinic in Atlanta, Georgia was conducted from July, 2013 to November, 2014 to evaluate factors associated with self-reported condom use. Primary outcomes included: condom use at last vaginal intercourse and consistent condom use with vaginal intercourse over the last six months. Descriptive, bivariable, and multivariable logistic regression analyses were performed.

**Results:**

Of 187 women enrolled, 170 reported having vaginal intercourse in the last six months. Seventy-four percent used condoms at last vaginal intercourse, whereas 53% reported consistent condom use over the last six months. In adjusted analyses, factors associated with condom use at last intercourse included decreased frequency of sex, no history of drug use, and confidence to discuss condom use with sexual partners (p<0.05). Factors associated with consistent condom use in the past six months were older age, being single/dating, and confidence to discuss condom use with sexual partners. History of drug use, having HIV-positive partners, and unprotected anal intercourse were associated with inconsistent use (p<0.05).

**Conclusions:**

Improved strategies are needed to educate women on the importance of safe sexual practices and condom negotiation. Healthcare providers should strive to have an open dialogue with patients about condom use, whether they engage in anal sex, and its risks.

## Introduction

Unprotected sexual intercourse accounts for the largest proportion of new HIV infections globally [1–2]. In 2016, adult and adolescent women comprised an estimated 19% of the 39,000 new HIV infections in the United States (U.S.), with 86% resulting from heterosexual contact [1]. Despite multiple studies showing that correct and consistent condom use can reduce STD and HIV transmission risk by 80%, actual rates of condom use in high risk and HIV-positive women remain suboptimal [3–5]. In a large, national sample of heterosexually active female and male adults ages 15–44, 21% of women reported using a condom with last sex compared to 29% of men [6]. Even more concerning, was the statistically significant finding that only 32% of single/unmarried women with HIV-related sexual risk behaviors reported using a condom, which included those having an HIV-positive partner [6]. It is well established that high-risk sexual behaviors increase the transmission of HIV but these risks are even greater in women who are already HIV positive, because they not only have the potential to infect sero-negative partners, but they themselves are also susceptible to other pathogens or resistant strains of HIV. As such, consistent condom use in HIV positive women is even more important because it serves as a measure of both primary and secondary prevention [3–5, 7].

Many of the previous studies that have examined condom use practices in HIV-positive adult women were done in sub-Saharan Africa where HIV rates are much higher compared to the U.S. [8–10]. However, these populations may not be generalizable due to socio-economic, cultural, and religious differences. Within the U.S., the Southern states accounted for more than half of new HIV diagnoses in women in 2017 [1]. Furthermore, within the Southern states lives the highest concentration of Black/African Americans, a population that is disproportionately affected by HIV (43% of new HIV diagnoses, 59% of new HIV diagnoses in women, and 42% of all people living with HIV) despite the fact that they comprise only 12% of the nation’s population [1,11]. Additionally, a smaller percentage of African Americans living with HIV received medical care in 2014 with even fewer achieving a suppressed viral load compared to other racial/ethnic groups [12–13]. Promoting condom use as an effective method to prevent HIV transmission and development of HIV resistance is especially important in populations that are less likely to receive HIV treatment. Structural level interventions like condom distribution programs have been shown to have greater success in reducing the risks of HIV when combined with individual-level interventions [14].

It is important to understand the attitudes, behaviors, relationship dynamics and barriers faced by HIV-positive women during sexual encounters to better educate and promote safe sexual practices. The objective of this study is to evaluate factors associated with self-reported condom use in a cohort of adult, predominantly African American women receiving HIV care in Atlanta, Georgia. We hypothesized that multiple factors influence condom use, including those related to HIV as well as relationship, communication, and behavioral factors.

## Materials and methods

### Study population and procedures

This study is a secondary analysis from a cross-sectional evaluation of reproductive health knowledge, attitudes, and practices among women receiving HIV care at The Grady Infectious Diseases Program (IDP) Clinic in Atlanta, Georgia, a large, free-standing HIV/AIDS outpatient facility that serves over 6,000 patients (~25% female) each year [15]. Human subjects research approval was obtained from the Emory Institutional Review Board and the Grady Research Oversight Committee (IRB00057649).

To be eligible for the study, women had to be HIV-positive, aged 18–45 years, sexually active within the last 6 months, and English speaking. A convenience sample of women was recruited from the clinic waiting room prior to appointments from July 2013 to November 2014. The aim was to recruit up to 200 women based on feasibility within the 16-month time period allotted for study recruitment. Potentially eligible women were identified via evaluation of the daily schedule. Interested women were brought to a private room to ensure eligibility and obtain written consent. Twenty-six percent of those approached were not interested in participating. We did not complete a participation log so detailed information about the reasons these women declined are unknown. Among those interested, 31% were ineligible because they were non-English speaking, not sexually active, or unable to consent.

Participants were invited to complete a 30-minute Audio Computer Assisted Self Interview (ACASI) questionnaire using a laptop computer and headphones. Trained research staff was available to answer questions if needed; however, the electronic survey format was developed to minimize social desirability biases and low literacy barriers. The questionnaire consisted of 225 items that assessed individual demographic, behavioral, and clinical characteristics, as well as relationship characteristics utilizing a social-ecological framework. The social-ecological framework is a theory-based framework for understanding how the interconnected factors of the individual and interpersonal relationships, community, relevant organizations, and larger environment influence behaviors and behavior change [16]. By incorporating this framework, we sought to examine the dynamic interplay between individual factors and environmental or social conditions that may influence condom use. The survey items reflect common themes and concerns previously expressed by focus groups of women at The Grady IDP Clinic and in the literature. All responses were self-reported. Upon survey completion, participants received a $5 gift card as compensation.

### Study outcomes

The primary outcomes were 1. Condom use at last vaginal intercourse (“The last time you had vaginal sex, did you use a condom?” with yes/no response options); and 2. Consistent condom use with vaginal intercourse over the last six months (“In the past 6 months, how often did you have vaginal sex with a condom?” with Likert scale response options). The Likert scale responses were collapsed into two categories—consistent use meant that the participant always used condoms with vaginal intercourse in the last six months while inconsistent use meant that condoms were not always used or not used at all.

Covariates of interest included: demographic variables (age, race, education level, and current employment status); STD history; relationship status (married, in a committed relationship, dating, or single); sexual behaviors (age of initial intercourse, number of sexual partners, frequency of intercourse, and engaging in anal intercourse); any history of alcohol or illicit drug use; plans to have children in the future (fertility intention); self-confidence to ask a male partner to use a condom even if he does not want to wear one; and variables related to HIV, including route of HIV acquisition, partner HIV sero-status, HIV status disclosure, self-reported viral load (detectable versus undetectable or unknown), and antiretroviral treatment (ART) use. Participant age was categorized by quartiles. Partners’ HIV sero-status was categorized as some or all HIV-positive partners versus none (HIV negative partners only).

A secondary objective was to determine the reasons why women did or did not use condoms the last time they had vaginal intercourse. Women responding “yes” to the question “The last time you had vaginal sex, did you use a condom?” were asked to select their reasons (all that apply) from the following options: “to prevent pregnancy,” “to prevent STDs,” or “to prevent HIV.” For women who responded “no,” a selection of twenty common reasons for non-use were listed, and respondents selected whether each reason played a “very important role”, “somewhat important role”, or “no role”. We also asked women about risks of transmitting HIV to their partners at the time of sex without condoms based on ART use/non-use.

### Data analytic approach

Data were analyzed using SPSS Version 21.0 (IBM Corp, Armonk, NY). All statistical tests were evaluated at the two-sided, 0.05 level of significance. The distributions of demographic, sexual behavior, and psychosocial characteristics of participants were summarized with frequencies. Pearson’s chi-square (or Fisher’s exact) tests evaluated associations between categorical covariates and the outcomes of interest. Independent variables that were significantly related (p<0.05) to the outcomes in bivariable analysis were included in multivariable logistic regression models. There were no collinear variables in the multiple logistic regression model based on a variance inflation factor greater than 10, a tolerance value less than 0.1, or a condition index greater than 30. Unadjusted prevalence odds ratios (PORs) and adjusted prevalence odds ratios (aPORs) with 95% confidence intervals (CIs) were calculated.

## Results

Of 187 women enrolled in the study, 170 reported having vaginal intercourse in the past six months and answered the questions pertaining to primary outcomes of interest. The mean age was 35.3 (standard deviation 7.6). Most participants were African-American, had an educational level of high school or less, and were unemployed (88%, 58%, and 66%, respectively; Tables 1 and 2). Two-thirds were married or in a committed relationship; over half of those women had been in their current relationship for at least one year.

**Table 1:** Demographics and associations with condom use at last vaginal intercourse among sexually active HIV-positive women (N = 170).

Characteristic	Row total	Condom use with last intercourse / (%)	Unadjusted POR (95% CI)	Adjusted POR^b^ (95% CI)
Age				
< 31	45	29 (64)	1 (Ref)	1 (Ref)
32–37	49	36 (73)	1.53 (0.63–3.68)	0.98 (0.34, 2.85)
38–42	36	26 (72)	1.43 (0.55–3.71)	0.72 (0.22, 2.32)
≥ 43	40	34 (85)	3.13 (1.08–9.03)^a^	1.44 (0.40, 5.14)
Race				
African American	149	108 (73)	1 (Ref)	
White/other	21	17 (81)	1.61 (0.51, 5.08)	
Education				
≤ HS	98	68 (69)	1 (Ref)	
> HS	72	57 (79)	1.68 (0.82, 3.42)	
Current Employment				
No	112	77 (69)	1 (Ref)	
Yes	58	48 (83)	2.18 (0.99, 4.81)	
Route of HIV acquisition				
Heterosexual sex	141	104 (74)	1 (Ref)	
Other	29	21 (72)	0.93 (0.38, 2.29)	
STD history				
No	75	59 (79)	1 (Ref)	
Yes	95	66 (69)	0.62 (0.31, 1.25)	
Worry about an STD				
No	139	108 (78)	1 (Ref)	1 (Ref)
Yes	31	17 (55)	0.35 (0.15, 0.79)^a^	0.39 (0.15, 1.04)
Relationship status				
Married/Committed	114	80 (70)	1 (Ref)	
Single/dating	56	45 (80)	1.74 (0.80, 3.76)	
Partner has sex w/others				
No	116	90 (78)	1 (Ref)	
Yes	54	35 (65)	0.53 (0.26, 1.08)	
Age of initial intercourse				
> 16	70	57 (81)	1 (Ref)	
≤ 16	100	68 (68)	0.48 (0.23, 1.01)	
Anal sex w/condom last 6mo				
Always	21	18 (85)	1 (Ref)	
Not Always	52	33 (63)	0.29 (0.08, 1.11)	
Never had anal sex	97	74 (76)	0.54 (0.15, 1.99)	
No. sexual partners last 6mo				
≤ 1	123	90 (73)	1 (Ref)	
> 1	47	35 (74)	1.07 (0.50, 2.30)	
Frequency of sex last 6mo				
< 1/week	97	79 (81)	1 (Ref)	1 (Ref)
≥ 1/week	72	45 (63)	0.38 (0.19, 0.76)^a^	0.44 (0.20, 0.97)^a^
No. partners known HIV+				
None	112	84 (75)	1 (Ref)	
Some/All	58	41 (71)	0.80 (0.40, 1.63)	
Hx sexual or physical abuse				
No	76	60 (78)	1 (Ref)	
Yes	93	65 (66)	0.62 (0.31, 1.26)	
Hx of alcohol use				
No	39	31 (79)	1 (Ref)	
Yes	130	94 (72)	0.67 (0.28, 1.60)	
Hx of illicit drug use				
No	88	72 (82)	1 (Ref)	1 (Ref)
Yes	81	53 (65)	0.42 (0.21, 0.85)^a^	0.43 (0.19, 0.98)^a^
Desires future pregnancy				
No	115	92 (80)	1 (Ref)	1 (Ref)
Yes	54	33 (61)	0.39 (0.19, 0.80)^a^	0.42 (0.17, 1.01)
Currently using birth control				
No	109	78 (72)	1 (Ref)	
Yes	59	46 (78)	1.41 (0.67, 2.96)	
Currently using ART				
No	44	31 (70)	1 (Ref)	
Yes	119	88 (74)	1.19 (0.55, 2.56)	
Most recent viral load				
Undetectable	109	81 (74)	1 (Ref)	
Detectable/unknown	61	44 (72)	1.11 (0.55, 2.26)	
HIV status disclosed				
None	35	27 (77)	1 (Ref)	
Some/All	135	98 (73)	0.78 (0.33, 1.88)	
Confidence to discuss condom use w/partner if he does not want to wear one				
No	31	16 (52)	1 (Ref)	1 (Ref)
Yes	130	102 (79)	3.42 (1.51, 7.75)^a^	3.88 (1.15, 9.64)^a^
Who makes decisions about condom use				
She does/Both do	146	110 (75)	1 (Ref)	
He does	14	8 (57)	0.44 (0.14, 1.34)	
Depression				
No	62	46 (74)	1 (Ref)	
Yes	98	72 (73)	0.96 (0.47, 1.99)	

^a^ Statistically significant variable with p<0.05

^b^ Statistically significant variables (p<0.05) in bivariable analysis were included in multivariable logistic regression models to calculate an adjusted prevalence odds ratio (aPOR)

*POR* prevalence odds ratio; *CI* confidence interval; *HS* high school; *ART* antiretroviral therapy

**Table 2:** Demographics and associations with consistent condom use during vaginal intercourse over last 6 months among sexually active HIV-positive women (N = 170).

Characteristic	Row total	Consistent condom use over last 6 months / (%)	Unadjusted POR (95% CI)	Adjusted POR (95% CI)^b^
Age				
< 31	45	17 (38)	1 (Ref)	1 (Ref)
32–37	49	27 (55)	2.02 (0.89, 4.60)	2.73 (0.97, 7.64)
38–42	36	18 (50)	1.65 (0.68, 4.01)	1.86 (0.61, 5.65)
≥ 43	40	28 (70)	3.84 (1.55, 9.51)^a^	4.67 (1.49, 14.61)^a^
Race				
African American	149	82 (55)	1 (Ref)	
White/other	21	8 (38)	0.50 (0.20, 1.29)	
Education				
≤ HS	98	48 (49)	1 (Ref)	
> HS	72	42 (58)	1.46 (0.79, 2.70)	
Current Employment				
No	112	57 (51)	1 (Ref)	
Yes	58	33 (57)	1.27 (0.67, 2.41)	
Route of HIV acquisition				
Heterosexual sex	141	74 (52)	1 (Ref)	
Other	29	16 (55)	1.11 (0.50, 2.49)	
STD history				
No	75	40 (53)	1 (Ref)	
Yes	95	50 (52)	0.97 (0.53, 1.78)	
Worry about an STD				
No	139	77 (55)	1 (Ref)	
Yes	31	13 (42)	0.58 (0.26, 1.28)	
Relationship status				
Married/Committed	114	51 (45)	1 (Ref)	1 (Ref)
Single/dating	56	39 (70)	2.83 (1.44, 5.59)^a^	2.39 (1.04, 5.50)^a^
Partner has sex w/others				
No	116	60 (52)	1 (Ref)	
Yes	54	30 (56)	1.17 (0.61, 2.23)	
Age of initial intercourse				
> 16	70	40 (57)	1 (Ref)	
≤ 16	100	50 (50)	0.75 (0.41, 1.39)	
Anal sex w/condom last 6mo				
Always	21	15 (71)	1 (Ref)	1 (Ref)
Not always	52	19 (37)	0.23 (0.48, 0.70)^a^	0.17 (0.04, 0.64)^a^
Never had anal sex	97	56 (58)	0.55 (0.20, 1.53)	0.46 (0.13, 1.58)
No. sexual partners last 6mo				
≤ 1	123	67 (54)	1 (Ref)	
> 1	47	23 (49)	0.80 (0.41, 1.57)	
Frequency of sex last 6mo				
< 1/week	97	58 (60)	1 (Ref)	1 (Ref)
≥ 1/week	72	32 (44)	0.54 (0.29, 0.997)^a^	0.95 (0.45, 2.00)
No. partners known HIV+				
None	112	68 (61)	1 (Ref)	1 (Ref)
Some/All	58	22 (38)	0.40 (0.21, 0.76)^a^	0.40 (0.18, 0.89)^a^
Hx sexual or physical abuse				
No	76	44 (54)	1 (Ref)	
Yes	93	46 (52)	0.71 (0.39, 1.31)	
Hx of alcohol use				
No	39	17 (44)	1 (Ref)	
Yes	130	73 (56)	1.66 (0.81, 3.41)	
Hx of illicit drug use				
No	88	54 (61)	1 (Ref)	1 (Ref)
Yes	81	36 (44)	0.50 (0.27, 0.93)^a^	0.34 (0.16, 0.72)^a^
Desires future pregnancy				
No	115	68 (59)	1 (Ref)	1 (Ref)
Yes	54	22 (41)	0.48 (0.25, 0.92)^a^	0.83 (0.35, 1.96)
Currently using birth control				
No	109	55 (50)	1 (Ref)	
Yes	59	35 (59)	1.43 (0.75, 2.72)	
Currently using ART				
No	44	21 (48)	1 (Ref)	
Yes	119	68 (57)	1.46 (0.73, 2.92)	
Most recent viral load				
Undetectable	110	58 (53)	1 (Ref)	
Detectable/unknown	61	33 (54)	1.06 (0.56, 1.98)	
HIV status disclosed				
None	35	19 (54)	1 (Ref)	
Some/All	135	71 (53)	0.93 (0.44, 1.97)	
Confidence to discuss condom use w/partner if he does not want to wear one				
No	31	11 (36)	1 (Ref)	1 (Ref)
Yes	130	77 (59)	2.64 (1.17, 5.97)^a^	2.62 (1.06, 6.50)^a^
Who makes decisions about condoms use				
She does/Both do	146	83 (57)	1 (Ref)	
He does	14	5 (36)	0.42 (0.13, 1.32)	
Depression				
No	62	33 (53)	1 (Ref)	
Yes	98	55 (56)	1.12 (0.59, 2.13)	

^a^ Statistically significant variable with p<0.05

^b^ Statistically significant variables (p<0.05) in bivariable analysis were included in multivariable logistic regression models to calculate an adjusted prevalence odds ratio (aPOR)

*POR* prevalence odds ratio; *CI* confidence interval; *HS* high school; *ART* antiretroviral therapy

Heterosexual transmission was the principal transmission mode (83%). Forty-two percent had engaged in anal sex in the last six months. The majority were currently taking ART (73%), had undetectable viral loads based on self report (64%), and did not have an AIDS diagnosis (58%). Over half reported first intercourse by age 16. Seventy-four percent used condoms the last time they had vaginal intercourse and 53% always used condoms over the last six months.

### Factors associated with condom use at last vaginal intercourse

As shown in Table 1, women of older age (≥43 years) had increased odds of condom use at last vaginal intercourse relative to women under 31 in unadjusted analyses (POR 3.13; CI 1.08, 9.03). Lack of confidence in asking male partners to use a condom when they did not want to (POR 0.29, CI 0.13, 0.66), increased frequency of sex (at least once per week) (POR 0.44; CI 0.20, 0.97), and history of illicit drug use (POR 0.43; CI 0.19, 0.98) were associated with not using a condom at last vaginal intercourse in unadjusted analyses. Although history of STDs was not associated with condom use, the odds of using condoms were less among those who felt worried about STD transmission from a partner in the past 6 months compared to those who had no concerns (POR 0.39; CI 0.15, 0.79). In adjusted analyses, confidence to discuss condom use with a partner (aPOR 3.88; CI 1.15, 9.64), frequency of sex in the last 6 months (aPOR 0.44; CI 0.20, 0.97), and previous illicit drug use (aPOR 0.42; 0.19, 0.98), remained significantly associated with condom use at last vaginal intercourse.

### Factors associated with condom use during vaginal intercourse over the past six months

As shown in Table 2, being 43 years of age or older was associated with consistent condom use (always using a condoms) during vaginal intercourse over the past six months compared to women under 31 (POR 3.84; CI 1.55, 9.51). Women who were single or casually dating (POR 2.83; CI 1.44, 5.59) or who reported feeling confident to discuss condom use with sexual partners (POR 2.64; CI 1.17, 5.97) had higher odds of always using condoms in unadjusted analyses. Of the 43% of women reporting anal sex in the last six months, only 29% always used condoms. Women who were inconsistent in condom use with anal sex had decreased odds of reporting protected vaginal sex when compared to women who always used condoms with anal sex (POR 0.23; CI 0.48, 0.70). Other behavioral factors associated with inconsistent condom use in unadjusted analyses were history of illicit drug use, fertility intention, and increased sexual frequency. Women who knew that some or all sexual partners during the past six months were HIV-positive also had decreased odds of always using condoms compared to women in sero-discordant relationships (POR 0.40; CI 0.21, 0.76). In multivariable analyses, the following variables remained significantly associated with consistent condom use in the past six months: older age; being single/dating; using condoms during anal intercourse; having no known HIV-positive sexual partners; having no history of illicit drug use; and confidence to discuss condom use with partners (Table 2, Adjusted POR).

### Reasons why women did or did not use condoms at last vaginal intercourse

Among the 125 women that used condoms at last vaginal intercourse, preventing HIV transmission was the most frequently selected motivating factor, followed by prevention of STDs and pregnancy (74%, 70%, 37%, respectively). Participants who reported not using condoms at last vaginal intercourse cited the following factors in their decision: did not think about it (52%); did not have a condom (61%); either she or her partner had recently tested negative for STDs (54%); she and her partner only had sex with each other (62%); and she and her partner trusted each other (58%).

Participant responses regarding the perceived risk of HIV transmission during unprotected sex were similar regardless of ART use. The majority of women believed that the risk was greater than 50% regardless of ART use (71% of women on ART and 83% of women not on ART). For those 187 women who responded that HIV transmission during unprotected intercourse was certain (100%) with or without ART, over 40% still did not use condoms consistently over the last six months.

## Discussion

Rates of condom use at last vaginal intercourse (74%) and consistent condom use (always) during vaginal intercourse in the past six months (53%) were suboptimal in our cohort of adult women, similar to findings in prior studies among adolescent HIV-positive females as well as a large multicenter study of HIV-positive and high-risk HIV-negative adult women in the U.S. [17–22]. Although women who used condoms during last vaginal intercourse cited HIV prevention as their main reason for condom use, the decreased rate of consistent condom use during the previous six-month period demonstrates that other factors influenced their actual practices over multiple sexual episodes. This is also supported by the trend toward reduced condom use in women who reported greater sexual frequency, which has been seen in other studies [10, 23].

Women in the oldest age group were almost five times as likely in adjusted analyses to report consistent condom use in the past six months versus women in the youngest group. Older women in our cohort may have been better able to negotiate condom use with sexual partners because of maturity, previous experiences, willingness to refuse unprotected sex, and lack of desire for future fertility [8]. Their behaviors contradict the findings of two large national surveys where the percentage of women having unprotected sex increased with age, but HIV sero-status was not taken into account [6, 24]. Our study did not reveal a difference in condom use by race, however, the majority of HIV-positive women (over 80%) in our population demographic identified as African American, which is consistent with the Centers for Disease Control and Prevention data that African American women are disproportionately affected by HIV [12].

Women who were single or dating had three times the odds of always having protected vaginal intercourse compared to their married/committed counterparts. This finding is similar to studies of at-risk heterosexual women in the U.S., including one that assessed predictors of unsafe sex in a group of at-risk women in Atlanta [25–27]. Women in urban communities have shown less intention of using condoms with “main” or steady sexual partners compared to secondary partners and they often defer decision-making to the main male partner [25–27]. Reasons for condomless sex with a main or committed partner were related to trust, infidelity, and fear of arousing suspicion, rejection or anger leading to abuse [25]. The most common reasons why women in our study reported not using condoms the last time they had sex were that they did not think about it, that they only had sex with each other, and that they trusted each other. In order to overcome the perceived barriers to condom use by women in committed relationships or with known HIV-positive partners, couples’ counseling programs that address relationship dynamics and promote equal decision-making power between heterosexual partners should be implemented per World Health Organization guidelines [28].

Self-confidence to negotiate condom use, even when a male partner does not want to wear one, was significantly associated with consistent condom use. Several studies have found an association between self-esteem levels and involvement in risky sexual behaviors, including unprotected intercourse due to antagonistic feelings about condom use and failure to disclose HIV or STD status to sexual partners [17–18, 26, 29–30]. Self-confidence and self-esteem are not mutually exclusive concepts; the ability to assert oneself in specific situations like negotiating condom use may be enhanced by having positive feelings about oneself [31]. The majority of women in our cohort (81%) reported self-confidence to discuss condoms and had revealed their HIV status to some or all of their sexual partners (79%). These findings further support the importance of: developing interventions to bolster women’s self-esteem to increase their likelihood of engaging in protected sex; and developing female-controlled modalities that do not require partner acquiescence or approval, which is especially important for women who are still working to acquire self-confidence. A meta-analysis by Crepaz et al revealed that in African American women specifically, behavioral interventions that addressed empowerment issues and negotiating skills pertaining to safe sex and condom use were successful in HIV/STD prevention [32]. Together, individual and couple’s counseling programs offer multiple forums in which women can address important personal and relationship dynamics that affect their ability to achieve higher levels of condom efficacy.

Women with only HIV-negative partners were more likely to use condoms consistently than women in sero-concordant relationships. Condom use still plays an integral role in preventing other STDs or HIV super-infections in concordant positive couples; thus, it is imperative that HIV-positive people be counseled on the value of using condoms with every sexual partner, regardless of their sero-status or ART use. Additionally, we wanted to see whether perceived viral load and ART use at the time of intercourse had any impact on condom use practices because there were concerns in earlier studies, mainly in homosexual men that ART would promote riskier sexual behaviors because of decreased HIV transmission risk [33–34]. However, there was no significant difference in consistent or recent condom use between women currently taking ART and those who were not, nor in women who reported having a detectable viral load [35]. Furthermore, results of the secondary descriptive analysis showed that our study population believed that the risk of HIV transmission is over 50% at the time of sex without condoms even with ART use, but they still did not use condoms consistently.

Finally, our results demonstrate an association between consistent condom use during vaginal intercourse and during anal intercourse within the last six months. Women engaging in protected anal intercourse were more likely to have protected vaginal intercourse. Unfortunately, the total number of women who always used condoms with anal intercourse was a small percentage (29%) of those who admitted to having anal intercourse at all, despite it being one of the riskiest sexual behaviors for infection transmission. Factors associated with condom use during anal intercourse have been assessed in men who have sex with men; however, less information is available for heterosexual, HIV-positive women [33–34]. Nasrullah’s national survey of heterosexually active women whose HIV serostatus was not reported demonstrated that the overall prevalence of condom use with anal sex was less than with vaginal sex and were affected by gender and marital status [6]. Reasons for decreased condom use with anal intercourse may be due to the absence of pregnancy risk for women, partner dynamics and communication about anal intercourse, and lack of knowledge pertaining to the risks of infection transmission with anal intercourse. Efforts should be made to evaluate anal sex risk in all women, and especially HIV-positive women, during routine screening for behavioral risk factors and provide appropriate education.

Our outcomes of interest are limited by recall bias. We relied on subjective retrospective reporting, which may be inaccurate given the need for participants to remember condom use during every coital episode. Previous studies have demonstrated that self-reported condom use may be an unreliable indicator of true condom use, and condom-failures were not considered [36]. This study may also be limited by selection bias because we used a convenience sample and did not obtain detailed information on patients who chose not to participate in the study. Finally, for our secondary analysis only, we had a different set of questions for participants who did not use condoms with last intercourse, which decreased the sample size of responses in these analyses. Our findings are most generalizable to HIV-positive, lower socio-economic status African American women living in the Southeast U.S. A strength of our study is that it broadens our understanding of factors associated with condom use in a population that has disproportionately higher rates of HIV and lower rates of treatment.

Empowering HIV-positive women to discuss consistent and correct condom use with sexual partners is critically important to prevent onward transmission. Health care providers should strive to have an open dialogue with patients about whether they engage in high-risk behaviors like anal sex or illicit drug use, the specific risks of anal sex, whether they feel comfortable negotiating condom use, and counsel on risk reduction strategies. Furthermore, dispelling misconceptions about HIV/STD transmission in HIV positive individuals with repetitive educational messaging and routine condom distribution may improve consistent condom use.

## Supporting information

S1 Dataset The S1 Dataset contains the de-identified data corresponding to the responses of study participant. (XLSX)

S1 Codebook The S1 Codebook contains the 225-item questionnaire that was completed by study participants. (DOCX)
