# Supplementary material for: Factors associated with condom use among HIV-positive women living in Atlanta, Georgia
Source: PLoS One. 2019 Dec 13;14(12):e0225406. doi: 10.1371/journal.pone.0225406 (PMC6910822; doi:10.1371/journal.pone.0225406)
Supplement: S1 Codebook — (DOCX) [file pone.0225406.s002.docx]

**PONCE QUANTITATIVE SURVEY**

**I. ACASI Instructions and Practice Questions (Section P)**

**Instructions**

*(To be read to participants, instructions will not appear as text.)*

**Inst1**. This private survey asks about behaviors that affect the health of HIV-infected women. We’re asking these questions to learn about your feelings, beliefs, behaviors and relationships. Your input really matters to us! To help us learn from your experiences, please tell us what you really think and what your actual experiences have been, not just what you think we’d want to hear. Remember, all your responses are kept private. Your name does not appear on this survey. If you find a question that you don’t understand or have difficulty completing a question, please let the research assistant know and a staff member will assist you. Also, if you answer something incorrectly and want to change an answer, let the research assistant know. Finally, let the research assistant know when you are completely finished with the survey. Thank you again.

Let’s get started!

# **Practice Questions**

**Inst2**. *First, we’re going to do a few practice questions so you can learn how this computer survey works.*

ii1. Some questions are going to ask you to select one response from several choices, so you would click one box. For example, if the question is, “What is your favorite color?”, you would click only one box for your response. Throughout the survey, please select the *best* answer for each of the questions.

1. Blue
2. Red
3. Green
4. Yellow

ii2. Some questions will ask that you check ALL the boxes that apply. For example, if the question is, “What pets have you had in your life?”, you would check the box for each different type of pet you have had. If you have never had any of these pets, you do not select any of the boxes. After you have answered the question, press continue in the bottom right hand corner to go to the next question.

1. Cat
2. Dog
3. Fish
4. Hamster

ii3. Other questions are going to ask that you provide a specific number for a response. For example, if the question is, “What is your favorite number?”, you would type in a number using the key pad. ##

ii4. After the directions for the section of a survey are read, you can press the “Continue” key in the lower right corner to move forward to the next question.

ii5. If you realize you might have checked the wrong box, please notify a research assistant before continuing with the questionnaire

ii6. If you have any questions during the survey, just let the research assistant know and they will help you. Okay, let’s get started with the survey! When you are ready to begin, click continue in the lower right hand corner of the screen. *Thanks for participating in this study!*

*First we are going to do a few practice questions so you can learn how this computer survey works.*

P1. Some questions are going to ask you to choose one response from a few choices, so you would click one box. For example, choose your answer to, "What is your favorite color?", by clicking one of the boxes below.

1. Blue
2. Red
3. Green
4. Yellow

P2. “What is your favorite number?”, type in a number using the keypad.

**Inst3**. *Now we will begin the questionnaire. Please do your best to answer all questions. If you are not sure of an answer, pick the answer that* most closely *describes you or your situation.*

**II. Demographics (Section A)**

A1. What is your gender?

1. Female
2. Male
3. Transgender

A2. How old are you?

_____ years old

A3. What is the highest level of education that you have completed?

1. Less than high-school
2. High-school diploma or GED
3. Some college
4. Associates degree or Technical Certification
5. Bachelors degree
6. Masters degree
7. Doctoral degree

A4. Which of the following BEST describes your racial/ethnic background?

1. Native Hawaiian or Other Pacific Islander
2. Black or African-American
3. White/Caucasian
4. Asian
5. American Indian or Alaska Native
6. Mixed or Multi-racial

A5. Do you consider yourself to be Hispanic/Latina/Latino?

1. No
2. Yes

A6. Is English your first language?

1. No
2. Yes

A7. Are you currently employed? (If value = No, skip to A9)

1. No
2. Yes

A8. On average, how many hours per week do you work?

_______ hours

A9. From which of the following sources do you regularly receive income? (Check all that apply)

- 1. Friends
  2. Wages or salary from job
  3. Unemployment
  4. Welfare
  5. Disability
  6. Spouse/Partner
  7. Family
  8. Other

A10. What is your health insurance status? (Check all that apply)

1. No insurance/self pay
2. Private health insurance or HMO (e.g. Blue Cross/ Blue Shield, Aetna)
3. Medicaid
4. Medicare
5. Other coverage (for example Ryan White or grant funding)
6. I don’t know

A11. Where do you live?

1. My own home or apartment
2. My family’s house or apartment
3. Someone else’s house or apartment (not family)
4. A rooming house or single room hotel
5. A shelter
6. A group home or halfway house
7. Other

**III. HIV Background (section B)**

**BInst1**. *The next set of questions is about HIV.*

B1. In what year were you diagnosed with HIV? (Give an estimated year if unsure)

_______

B2. How old were you when you first learned that you were HIV+? (Give an estimated age if unsure; if you were born with HIV, enter 0)

______ years old

B3. What was your most recent viral load result?

1. Undetectable
2. Detectable
3. Don’t know

B4. What was your CD4 or helper t-cell count in your MOST RECENT test report? If you are unsure of the exact number, please guess as closely as you can.

1. <50
2. 50-200
3. 200-500
4. >500
5. Unsure

B5. Have you ever been hospitalized for an HIV-related illness?

1. No
2. Yes

B6. Have you been diagnosed with AIDS?

1. No
2. Yes
3. Don’t know

B7. What is the most likely way that you became infected with HIV? Please check all that apply.

1. Sex with a man who was HIV+
2. Sex with a woman who was HIV+
3. Sharing needles
4. Blood transfusion
5. At birth (from my mother)
6. Other (Needle stick at work, etc.)
7. I don’t know how I was infected

**IV. STI Background (Section C)**

**CInst1**. *Now we are going to ask you about sexually transmitted infections, also known as STIs or STDs.*

C1. In the past 6 months, did you worry about getting an STD (sexually transmitted disease)?

1. No
2. Yes

C2. Have you *ever* been diagnosed or treated for one or more of the following? *Please check all that apply.* (If value=12, skip to C4)

1. Syphilis
2. Chlamydia
3. Gonorrhea
4. HPV (Genital warts)
5. Genital Herpes
6. Hepatitis A
7. Hepatitis B
8. Hepatitis C
9. Trichomonas
10. Other STD (Sexually transmitted disease)
11. I have been diagnosed/treated for an STD but don’t know which one
12. I have never been diagnosed with an STD

C3. Have you been diagnosed or treated for one or more of the following in the last *6 months*? Please check all that apply.

1. Syphilis
2. Chlamydia
3. Gonorrhea
4. HPV (Genital warts)
5. Genital Herpes
6. Hepatitis A
7. Hepatitis B
8. Hepatitis C
9. Trichomonas
10. Other STD (Sexually transmitted disease)
11. I have been diagnosed/treated for an STD in the last 6 months but don’t know which one
12. I have not been diagnosed with an STD in that last 6 months

**V. Sexual History and Current Sexual Behavior (Section D)**

**DInst1**. *The next series of questions is about sexual behavior. Most questions are about your current or most recent sexual partner.*

D1. Which best describes your current relationship status? (If value 3-5, skip to D3)

1. Married
2. In a committed relationship with only one partner
3. Dating one or more people
4. Single and interested in dating but not currently dating
5. Single and not interested in a relationship at all

D2. How long have you been with your *current* partner?

1. 3 months or less
2. 3-6 months
3. > 6 months to 1 year
4. >1 yr to 2 years
5. >2 yrs to 4 years
6. >4 years

D3. Can you describe your relationship with your *most recent* sexual partner (someone you have oral, vaginal or anal sex with)?

1. Marital partner
2. Other regular partner
3. Casual acquaintance
4. Some you just met
5. Commercial sex worker
6. Other

D4. Do you think your current sexual partner has had sex with other people since you started having sex together? If you are no longer in a relationship, do you think that your most recent sexual partner had sex with another person when you were still together?

1. Yes, I know for sure
2. I think so but I’m not sure
3. I don’t think so
4. No, I know for sure that he hasn’t

**DInst2**. *For the next group of questions we will be asking you about sexual activity.*

*When question says “any kind of sex,” it is asking about any and all of the following acts: oral sex, vaginal sex and anal sex. Any other time, the question will specify the type of sexual behavior being referred to. By sexual partners we mean someone you had oral, vaginal, or anal sex.*

*For questions that ask for a number, if you are unsure of the exact number, please give an estimated number.*

D5. Have you ever had oral sex? (if value=1(No), skip to D7)

1. No
2. Yes

D6. How old were you the first time you had oral sex?

_______ years old

D7. Have you ever had vaginal sex? (If value=1 (0 times), skip to D10)

1. No
2. Yes

D8. How old were you the first time you had vaginal sex?

_______ years old

D9. Old D12. In the past *6 months*, how often did you have ***vaginal*** sex ***with*** a condom?

1. Never
2. Less than half the time
3. About half the time
4. More than half the time but not always
5. Always

D10. Have you ever had anal sex? (if value=1(No), skip to D13)

1. No
2. Yes

D11. How old were you the first time you had anal sex?

_______ years old

D12. In the past 6 months, how often did you have **anal** sex **with** a condom?

1. Never
2. Less than half the time
3. About half the time
4. More than half the time but not always
5. Always

D13. How many ***male*** sexual partners have you ***ever*** had? *Count every person you’ve ever been with, even those you had any kind of sex with only once.(* If value=1 (0 partners), skip to D15)

1. 0 male partners
2. 1 male partner
3. 2-4 male partners
4. 5-10 male partners
5. 11-25 male partners
6. More than 25 male partners

D14. In the past ***6 months***, how many **male** sexual partners have you had? *Count every person, even those you had any kind of sex with only once.*

1. 0 male partners
2. 1 male partner
3. 2-4 male partners
4. 5-10 male partners
5. 11-25 male partners
6. More than 25 male partners

D15. How many ***female*** sexual partners have you ***ever*** had? *Count every person you’ve ever been with, even those you had any kind of sex with only once.*

1. 0 female partners
2. 1 female partner
3. 2-4 female partners
4. 5-10 female partners
5. 11-25 female partners
6. More than 25 female partners

D16. In the past *6 months*, how frequently have you had any kind of sex?

1. Only one or two times in the last 6 months
2. less than once per month
3. 1-2 times per month
4. About once a week
5. Several times a week

D17. Of the partners you have had in the past month, how many of your sexual partners knew that you are HIV positive?

1. None
2. Some
3. All

D18. During the past 6 months, how many of your sexual partners did you know were HIV positive?

1. None
2. Some
3. All

D19. Was/is your *most recent* sexual partner HIV positive?

1. No
2. Yes
3. I don’t know

D20. Was/is your most recent sexual partner aware of your HIV status?

1. No
2. Yes

D21. Old D26. The LAST time you had **vaginal** sex, did you use a condom? (If value=1(No), skip to D23A; if value=2(Yes), skip 28 & 29)

1. No
2. Yes

D22. what was/were the main reason(s) you used a condom the last time you had vaginal sex? *Select all that apply.*

1. To prevent pregnancy
2. To prevent stds
3. To prevent HIV

**DInst3**. *We are now going to give you some reasons people give for not using condoms. Please tell us if the given reason played a very important role, a somewhat important role, or no role in why you did not use a condom the last time you had sex?*

|  | **Reason condom not used** | **Influence level on condom use at last sex** | | |
| --- | --- | --- | --- | --- |
| D23A | we didn’t think about it | Very important (1) | Somewhat important (2) | Not important (3) |
| D23B | we didn’t have a condom | Very important (1) | Somewhat important (2) | Not important (3) |
| D23C | Too expensive | Very important (1) | Somewhat important (2) | Not important (3) |
| D23D | Ashamed to buy condoms | Very important (1) | Somewhat important (2) | Not important (3) |
| D23E | One (or both) of us is allergic to them | Very important (1) | Somewhat important (2) | Not important (3) |
| D23F | They break or slip | Very important (1) | Somewhat important (2) | Not important (3) |
| D23G | They are not good at preventing pregnancy | Very important (1) | Somewhat important (2) | Not important (3) |
| D23H | One (or both) of us wanted to get pregnant | Very important (1) | Somewhat important (2) | Not important (3) |
| D23I | I’m using other birth control | Very important (1) | Somewhat important (2) | Not important (3) |
| D23J | My partner refused | Very important (1) | Somewhat important (2) | Not important (3) |
| D23K | I did not want to use one | Very important (1) | Somewhat important (2) | Not important (3) |
| D23L | We did not talk about it | Very important (1) | Somewhat important (2) | Not important (3) |
| D23M | One or both of us had a negative std test | Very important (1) | Somewhat important (2) | Not important (3) |
| D23N | I was ashamed/afraid to ask to use one | Very important (1) | Somewhat important (2) | Not important (3) |
| D23O | we trust each other | Very important (1) | Somewhat important (2) | Not important (3) |
| D23P | we only have sex with each other | Very important (1) | Somewhat important (2) | Not important (3) |
| D23Q | We are both HIV positive | Very important (1) | Somewhat important (2) | Not important (3) |
| D23R | Condoms reduce sexual pleasure | Very important (1) | Somewhat important (2) | Not important (3) |
| D23S | Religious reasons 45 | Very important (1) | Somewhat important (2) | Not important (3) |
| D23T | we don’t like them for reasons other than those in the previous questions | Very important (1) | Somewhat important (2) | Not important (3) |

D24. How often do you use condoms?

1. More often than I would like
2. As often as I would like
3. Less often than I would like

D25. Have you ever used two condoms at the same time?

1. No
2. Yes

D26. Have you ever used the female condom?

1. No
2. Yes

D27. Old C20. Have you ever been in a relationship where you wanted to use a condom but your partner refused or threatened you? (Such as threatened to divorce/leave you)

1. No
2. Yes

D28. At any time in your life, has someone ever forced you to have oral, vaginal, or anal sex against your will? (If value=1(No), Skip to D30)

1. No
2. Yes

D29. In the past 6 months, has someone ever forced you to have oral, vaginal, or anal sex against your will?

1. No
2. Yes

D30. At any time in your life, has someone physically abused you (beat, punched, slapped)? (If value=1(No), Skip to D33)

1. No
2. Yes

D31. In the last 6 months, has someone physically abused you (beat, punched, slapped)?

1. No
2. Yes

D32. At any time in your life, has someone physically abused you because of your HIV status?

1. No
2. Yes

D33. Have you ever had sex in exchange for money, food, drugs, or a place to stay? (if value=1(No), skip to D35)

1. No
2. Yes

D34. In the past month, have you had sex in exchange for money, food, drugs, or a place to stay?

1. No
2. Yes

**DInst4**. *The next questions are about drinking alcohol and using drugs. Remember, all your answers are confidential. 57*

D35. Have you ever drunk any alcohol? (If value=1(No), Skip to D41) 58

1. No
2. Yes

D36. In the past 6 months, how often did you drink alcohol before or during sex?

1. Never
2. Less than half the time
3. About half the time
4. More than half the time but not always
5. Always

D37. Have you ever done any drugs? (If value=1(No), skip to Section E)

1. No
2. Yes

D38. In the past 6 months, how often did you use drugs before or during sex? (If value=1 (never), skip to Section E)

1. Never
2. Less than half the time
3. About half the time
4. More than half the time but not always
5. Always

D39. In the past 6 months, what drug(s) did you use prior to having sex? (Check all that apply)

1. Marijuana
2. Cocaine/Crack
3. Crystal Meth
4. Methamphetamines/Speed
5. Prescription medications not prescribed to you (ex: oxycontin, xanax, valium, etc.)
6. Other

**VI. Fertility preferences and pregnancy history** (Section E)

**EInst1**. *We are now going to ask about pregnancy and* *pregnancy related topics.*

E1. Have you ever been pregnant? (If value=1(no), skip to E19)

1. No
2. Yes

E2. How many children do you have?

1. No children
2. One
3. Two
4. Three
5. Four
6. Five or more children

E3. How old were you the FIRST time you got pregnant?

____ years old

E4. Have you ever had a miscarriage or delivered a stillborn?

1. No
2. Yes

E5. Were you ever HIV-positive during a pregnancy? (If value = 1(No), skip to E12)?

1. No
2. Yes

E6. The last time you became pregnant, did you know you were HIV-positive *before* you became pregnant?

1. No
2. Yes

E7. Did you find out you were HIV-positive during your most recent pregnancy?

1. No
2. Yes

E8. Did you take HIV medications during pregnancy? (If value = 1(No), skip to E11)?

1. No
2. Yes

E9. Did you stop taking HIV-medicines after the pregnancy?

1. No
2. Yes

E10. Did you breastfeed your baby?

1. No
2. Yes

E11. Have you ever had a child born with HIV?

1. No
2. Yes

E12. Have any of your children died due to HIV/AIDs?

1. No
2. Yes

E13. Have you ever had an unplanned pregnancy (a pregnancy that was mistimed, unplanned, or unwanted when you got pregnant)? (If value=1(no), skip to E15)

1. No
2. Yes

E14. How many unplanned pregnancies have you had?

________ unplanned pregnancies

E15. Have you ever had an abortion in the past (for any reason)? (If value=no, skip to E19)

1. No
2. Yes

E16. Have you ever had an abortion because of your HIV status?

1. No
2. Yes

E17. What was your partner’s HIV status for the pregnancy (or pregnancies) you aborted?

1. HIV-negative
2. HIV-positive
3. I have ended more than one pregnancy and partners have been both HIV positive and negative
4. I don’t know

E18. Did your partner’s HIV status influence your ending the pregnancy?

1. No
2. Yes

E19. In the past 6 months, did you worry about getting pregnant?

1. No
2. Yes

E20. Do you want or plan to have more children (at any time in the future)? (If no, skip to E22)

1. No
2. Yes

E21. Do you want or plan to have a child in the next two years?

1. No
2. Yes

E22. How much of a problem would it be if you were pregnant in the next 6 months?

1. It would be the worst thing that could happen
2. It would be a problem, but I’d eventually deal with it
3. It would not e a problem
4. I’d be happy about it

E23. Did getting diagnosed with HIV change whether you wanted or planned to have children?

1. No
2. Yes

E24. . Since being diagnosed with HIV, has your sexual desire changed?

1. Greater sexual desire
2. Same
3. Less sexual desire

E25. If you were HIV-negative, would you want to have more children?

1. No
2. Yes

E26. In the past 6 months, did you think you might not be able to get pregnant or might be infertile due to being HIV-positive?

1. No
2. Yes

E27. Does/did your most recent sexual partner want (more) children?

1. No
2. Yes

E28. Do/did you feel pressure to have more children from your most recent partner?

1. No
2. Yes

E29. Do you feel pressure to have more children from your family/community?

1. No
2. Yes

E30. Do you feel that getting pregnant would be too stressful on your health/body? (Will pregnancy be bad for your health?)

1. No
2. Yes

E31. In your opinion, does your community disapprove of HIV positive women having children?

1. No
2. Yes

E32. In your opinion, is it everyone’s right to have a child, regardless of HIV status?

1. No
2. Yes

E33. How do you feel about HIV-positive women getting pregnant if the risk of giving HIV to the baby is 100% (the baby will definitely be born with HIV)?

1. Strongly approve
2. Approve
3. Neutral
4. Disapprove
5. Strongly disapprove

E34. How do you feel about women getting pregnant if the risk of giving HIV to the baby is 50%?

1. Strongly approve
2. Approve
3. Neutral
4. Disapprove
5. Strongly disapprove

E35. How do you feel about women getting pregnant if the risk of giving HIV to the baby is 10%?

1. Strongly approve
2. Approve
3. Neutral
4. Disapprove
5. Strongly disapprove

E36. How do you feel about women getting pregnant if the risk of giving HIV to the baby is 1%?

1. Strongly approve
2. Approve
3. Neutral
4. Disapprove
5. Strongly disapprove

E37. Can HIV positive women give birth to HIV negative babies?

1. No
2. Yes

E38. Do you know about the services/treatments that help prevent mother to child transmission of HIV?

1. No
2. Yes

**EInst2**. *Now we would like to know about your discussions about contraception and sex with your HIV care providers and other health care workers.*

*Remember, your answers will not be shared with any of your providers. We want to know how you truly feel about your discussions about family planning with your HIV care providers.*

E39. Have you ever had a discussion about pregnancy with a health care worker since your HIV diagnosis? (if value=1(no), skip to E41)

1. No
2. Yes

E40. In the past year, have you discussed pregnancy with your doctor?

1. Yes
2. No

E41. Do you feel like you can discuss pregnancy with your doctor?

1. Yes
2. No

E42. In the past year, have you discussed contraceptives with your doctor?

1. Yes
2. No

E43. Do you feel like you can discuss contraceptives with your doctor?

1. Yes
2. No

E44. Do you feel that health care workers have given you enough information to make informed decisions about pregnancy since you were diagnosed with HIV?

1. No
2. Yes

E45. Have you felt that health care workers try to discourage you from having children after learning that you have HIV?

1. No
2. Yes

E46. Do you feel that you would have the support of your doctor if you decided to have more children?

1. No
2. Yes

E47. Do you feel that health care workers have given you enough information to make informed decisions about what contraceptives to use?

1. No
2. Yes

**Factors contributing to fertility preferences**

**EInst4**. *The next set of questions are about what factors influence whether or not you want to get pregnant and have a baby. For each item, indicate whether that factor has a strong, moderate, or no influence on you wanting to get pregnant.*

|  | **Item** | **Strong influence (1)** | **Moderate influence (2)** | **No influence (3)** |
| --- | --- | --- | --- | --- |
| E48. | Having a baby would be a big financial burden | Strong influence (1) | Moderate influence (2) | No influence (3) |
| E49. | Age influences whether or not I want to have a baby | Strong influence (1) | Moderate influence (2) | No influence (3) |
| E50. | The number of children I have influences whether or not I want to have a baby | Strong influence (1) | Moderate influence (2) | No influence (3) |
| E51. | My partner’s HIV status influences whether or not I want to have a baby | Strong influence (1) | Moderate influence (2) | No influence (3) |
| E52. | The desire to be a mom influences whether or not I want to have a baby | Strong influence (1) | Moderate influence (2) | No influence (3) |
| E53. | Worrying about passing HIV onto my baby influences whether or not I want to have a baby | Strong influence (1) | Moderate influence (2) | No influence (3) |
| E54. | Knowing an HIV-positive woman can have an HIV-negative baby influences whether or not I want to have a baby | Strong influence (1) | Moderate influence (2) | No influence (3) |
| E55. | Taking care of a baby would get in the way of my plans for the future | Strong influence (1) | Moderate influence (2) | No influence (3) |
| E56. | Taking care of a baby would be too much work for me | Strong influence (1) | Moderate influence (2) | No influence (3) |
| E57. | Worrying that if I pass away after my child is born, no one would take care of my child influences whether or not I want to have a baby | Strong influence (1) | Moderate influence (2) | No influence (3) |
| E58. | Worrying about passing the HIV to my partner in order to have more children influences whether or not I want to have a baby | Strong influence (1) | Moderate influence (2) | No influence (3) |
| E59. | Having a baby would be a way for me to hide my HIV status | Strong influence (1) | Moderate influence (2) | No influence (3) |
| E60. | I believe that some women have children to hide their own HIV status | False (1) | True (2) |  |

**VII. Contraceptive History (**Section F)

**FInst1**. *Now we are going to ask about what types of birth control methods (also known as contraceptives), if any, you have used and your experiences with them.*

F1. Have you ever used birth control? (By birth control we mean using something to not get pregnant (such as the Depo shot, pills, patches, rings, condoms, withdrawal, tubal ligation, etc.)? (If value = 1(No), skip to next section [Section G])

1. No
2. Yes

F2. Are you *currently* using any birth control?

1. No
2. Yes

F3. were you using any method of birth control (other than condoms) the last time you had sex? (If value=1(no), then skip to F5A)

1. No
2. Yes

F4. what methods of birth control (other than condoms) did you use the last time you had sex? *Check all that apply.* (Skip to F6 after this question)

1. Mirena IUD
2. ParaGuard IUD
3. Implant (Implanon)
4. Pill
5. Patch
6. Depo Provera (shot)
7. Ring
8. withdrawal
9. Emergency Contraception
10. Female sterilization (tubal ligation, had tubes tied)
11. Male sterilization (vasectomy)
12. Other

**FInst2**. *We are now going state some reasons people give for not using birth control. Please tell us if the given reason played a very important role, a somewhat important role, or no role in why you did not use a birth control method (other than condoms) the last time you had sex.*

|  | **Reason Birth control not used at last sex** | **Influence level on birth control use** | | |
| --- | --- | --- | --- | --- |
| F5A | I did not have a prescription/ my prescription ran out | Very important (1) | Somewhat important (2) | Not important (3) |
| F5B | Transportation problems | Very important (1) | Somewhat important (2) | Not important (3) |
| F5C | Too expensive | Very important (1) | Somewhat important (2) | Not important (3) |
| F5D | I tried birth control and didn’t like it | Very important (1) | Somewhat important (2) | Not important (3) |
| F5E | Other people told me not to use it | Very important (1) | Somewhat important (2) | Not important (3) |
| F5F | *I* wanted to get pregnant | Very important (1) | Somewhat important (2) | Not important (3) |
| F5G | *My partner* wanted me to get pregnant | Very important (1) | Somewhat important (2) | Not important (3) |
| F5H | I was worried about side effects | Very important (1) | Somewhat important (2) | Not important (3) |
| F5I | we used condoms | Very important (1) | Somewhat important (2) | Not important (3) |
| F5J | I wasn’t planning on having sex | Very important (1) | Somewhat important (2) | Not important (3) |
| F5K | Religious reasons | Very important (1) | Somewhat important (2) | Not important (3) |

F6. Do any medications you take play a role in deciding whether or not you use birth control?

1. Yes
2. No
3. Not applicable (I am not on any medications)

F7. Have you ever been in a relationship where you wanted to use birth control but your partner refused or threatened you (such as threatened to divorce you or physically hurt you)?

1. No
2. Yes

F8. Have health care providers ever discussed using condoms on top of another birth control method (also known as dual protection)?

1. No
2. Yes

F9. Did being diagnosed with HIV change how important you think it is to use a family planning/birth control method?

1. More important to use a family planning method
2. Same important
3. Less important

F10. which of the following did you do when deciding to use your last birth control method? *Pick all that apply.*

1. Talked to a doctor, nurse or health educator
2. Got information from magazines, TV, the internet, school or books
3. Talked to the guy you were having sex with about what he thought
4. Thought about how good the method is at preventing pregnancy
5. None of these, I really didn’t think about it at all

**FInst3**. *We are now going to ask you about what influences what type of family planning methods you choose. Please tell us if the given reason is a very important, somewhat important, or not important characteristic of a family planning method.*

|  | **Factors influencing use of birth control** | **Influence level on choice to use a birth control method** | | |
| --- | --- | --- | --- | --- |
| F11A | Effective at preventing pregnancy | Very important (1) | Somewhat important (2) | Not important (3) |
| F11B | Effective at preventing STIs/HIV transmission | Very important (1) | Somewhat important (2) | Not important (3) |
| F11C | Price | Very important (1) | Somewhat important (2) | Not important (3) |
| F11D | No one knows you are using it (it’s private) | Very important (1) | Somewhat important (2) | Not important (3) |
| F11E | Easy to use | Very important (1) | Somewhat important (2) | Not important (3) |
| F11F | Does not require a prescription | Very important (1) | Somewhat important (2) | Not important (3) |
| F11G | Partner is ok with it | Very important (1) | Somewhat important (2) | Not important (3) |
| F11H | Does not have hormones | Very important (1) | Somewhat important (2) | Not important (3) |
| F11I | I still get my period with I’m using it | Very important (1) | Somewhat important (2) | Not important (3) |
| F11J | It makes my periods get better or go away | Very important (1) | Somewhat important (2) | Not important (3) |
| F11K | Few/no side effects | Very important (1) | Somewhat important (2) | Not important (3) |
| F11L | I don’t have to do anything special to remember it | Very important (1) | Somewhat important (2) | Not important (3) |
| F11M | If I change my mind about having children, I can stop using it | Very important (1) | Somewhat important (2) | Not important (3) |
| F11N | I am in control of choosing to use it | Very important (1) | Somewhat important (2) | Not important (3) |
| F11O | Other people think it is a good method | Very important (1) | Somewhat important (2) | Not important (3) |

OCPs

F12. Old F28. Have you ever used the pill? (If value = 1(No), skip to F17 [Depo provera])

1. No
2. Yes

F13. when you used the pill, did you experience any of the following? *Pick all that apply.*

1. Periods got worse
2. Periods got better
3. Periods went away
4. Headaches, mood changes or nausea
5. Weight gain
6. Hair loss
7. Improved skin (Less acne)
8. Didn’t have any of these

F14. Did you use the pill in the last month? (If value = 1(no), skip to F17)

1. No
2. Yes

F15. In the past month, have you taken a pill every day? (If value = 2(yes), skip to F17)

1. No
2. Yes

F16. In the past month, how many pills did you *not* take (on time)?

_____ pills

DEPO PROVERA

F17. Have you ever used Depo Provera (shot)? (If value = 1(No), skip to F21 [Patch])

1. No
2. Yes

F18. when you used Depo, did you experience any of the following? *Pick all that apply.*

1. Periods got worse
2. Periods got better
3. Periods went away
4. Headaches, mood changes or nausea
5. weight gain
6. Hair loss
7. Improved skin (Less acne)
8. Didn’t have any of these

F19. Did you get you Depo shots when you were scheduled to?

1. Always
2. Sometimes
3. Rarely
4. I only got one shot

F20. Did you use Depo in the past month?

1. No
2. Yes

PATCH

F21. Have you ever used the birth control patch? (If value = 1(No), skip to F25 [Nuva Ring])

1. Yes
2. No

F22. when you used the patch, did you experience any of the following? *Pick all that apply.*

1. Periods got worse
2. Periods got better
3. Periods went away
4. Headaches, mood changes or nausea
5. weight gain
6. Hair loss
7. weight loss
8. Improved skin (Less acne)
9. Didn’t have any of these

F23. Did you use the patch in the past month? (If value = 1(No), skip to F25)

1. No
2. Yes

F24. During the past month, did you have a patch on every day except when you had your period?

1. No
2. Yes

NUVA RING

F25. Have you ever used the vaginal ring (Nuva Ring)? (If value = No, skip to F29 [Implanon])

1. No
2. Yes

F26. when you used the ring, did you experience any of the following? *Pick all that apply.*

1. Periods got worse
2. Periods got better
3. Periods went away
4. Headaches, mood changes or nausea
5. Weight gain
6. Hair loss
7. Weight loss
8. Improved skin (Less acne)
9. Didn’t have any of these

F27. Did you use the ring in the past month? (If value = 1(No), skip to F29)

1. No
2. Yes

F28. During the past month, did you have a ring in every day except when you had your period?

1. No
2. Yes

IMPLANON

F29. Have you ever used a contraceptive implant (Implanon or Nexplanon)? (If value = 1(No), skip to F32 [Paraguard])

1. No
2. Yes

F30. when you had the implant, did you experience any of the following? *Pick all that apply.*

1. Periods got worse
2. Periods got better
3. Periods went away
4. Headaches, mood changes or nausea
5. Weight gain
6. Hair loss
7. Weight loss
8. Improved skin (Less acne)
9. Didn’t have any of these

F31. Did you use the implant in the past month? (If value=1(no), go to F32)

1. No
2. Yes

PARAGUARD

F32. Have you ever used an IUD? (If value = 4 or 5 ( don’t know which or never used), skip to F37 [Female sterilization]; If value = 1(Mirena only), skip to F35 [Mirena])

1. Mirena IUD only
2. Paraguard Copper IUD only
3. Both Mirena and Paraguard
4. I have used an IUD but don’t know which kind
5. I have never used an IUD

F33. when you had ParaGuard, did you experience any of the following? *Pick all that apply.*

1. Periods got worse
2. Periods got better
3. Periods went away
4. Headaches, mood changes or nausea
5. Weight gain
6. Hair loss
7. Weight loss
8. Improved skin (Less acne)
9. Didn’t have any of these

F34. Did you use ParaGuard in the past month? (If F32 value=2(paraguard only), skip to F38)

1. No
2. Yes

MIRENA

F35. when you used Mirena, did you experience any of the following? *Pick all that apply.* (If value=10, skip to F37)

1. Periods got worse
2. Periods got better
3. Periods went away
4. Headaches, mood changes or nausea
5. Weight gain
6. Hair loss
7. Weight loss
8. Improved skin (Less acne)
9. Didn’t have any of these
10. Have never used Mirena

F36. Did you use Mirena in the past month?

1. No
2. Yes

FEMALE STERILIZATION

F37. Have you ever had a tubal ligation (also known as female sterilization or getting your tubes tied)? (If value = 1(No), skip to F42 [Withdrawal])

1. No
2. Yes

F38. How old were you when you had a tubal ligation? (Give an estimated year if unsure)

_______ years old

F39. Do you regret having a tubal ligation?

1. No
2. Yes

F40. Did you feel pressure from other people to have a tubal ligation?

1. No
2. Yes

F41. Was being HIV-positive an important factor in deciding to have a tubal ligation?

1. No
2. Yes
3. I was diagnosed with HIV after I had gotten a tubal ligation

WITHDRAWAL

F42. The next set of questions is about the withdrawal method, also known as the “pull out method,” where a guy pulls his penis out before he ejaculates. Have you ever used the withdrawal to prevent pregnancy? (If value = 1(No), skip to F44 [Other])

1. No
2. Yes

F43. Did you use withdrawal in the past month?

1. No
2. Yes

OTHER

F44. Have you ever used any of these other methods of birth control? *Pick all that apply*. (If value=6, skip to F91)

1. Diaphragm
2. spermicides
3. Sponge
4. Emergency Contraception (morning after pill)
5. Abortion
6. My partner had a vasectomy
7. I haven’t used any of these

F45. Have you ever used any of these other methods in the last month? *Pick all that apply.*

1. Diaphragm
2. spermicides
3. Sponge
4. Emergency Contraception (morning after pill)
5. Abortion
6. My partner had a vasectomy
7. I haven’t used any of these

**VIII. Contraceptive knowledge/attitudes (**section G)

**GInst1**. *With this next set of questions we are going to assess the general level of knowledge and misunderstandings about contraceptives. Not all statements are true, just answer to the best of your ability. The questions are mostly yes/no or true/false.*

G1. Have you heard of any birth control methods having a negative impact on your disease (worsening your HIV)? (If value = 1(No), skip to G3)

1. No
2. Yes

g2. Which birth control method(s) have you heard of having a negative impact on your disease (worsening your HIV)? *Pick all that apply.*

1. Depo Provera
2. Pills
3. IUD
4. Implant (Implanon)
5. Ring
6. Patch
7. None of the above

g3. Which birth control method(s) have you heard that you should not use when taking HIV medications? *Pick all that apply.*

1. Depo Provera
2. Pills
3. IUD
4. Implant (Implanon)
5. Ring
6. Patch
7. None of the above

G4. Have you heard of any birth control methods making it harder to get pregnant in the future? (If value = 1(No), skip to G6)

1. No
2. Yes

g5. Which birth control method(s) have you heard make it harder to get pregnant in the future? *Pick all that apply.*

1. Depo Provera
2. Pills
3. IUD
4. Implant (Implanon)
5. Ring
6. Patch
7. None of the above

G6. Before today, had you heard about the IUD (sometimes called the Mirena or ParaGuard)? (If value=1(no), skip to G8)

1. No
2. Yes

G7. Can a woman use an IUD even if she has never had a child?

1. No
2. Yes

G8. Before today, had you heard about the contraceptive implant, also known as Implanon or Nexplanon?

1. No
2. Yes

G9. Long acting methods like the implant or IUD can be removed early if a woman changes her mind and wants to become pregnant

1. No
2. Yes

G10. If you are using a birth control method, do you think you also need to use a condom?

1. No
2. Yes

G11. Do all hormonal birth control methods cause weight gain?

1. No
2. Yes

G12. It is ok to take birth control that stops your period from coming.

1. No
2. Yes

G13. IUDs move around in a woman’s body.

1. False
2. True

G14. women should take a break from hormonal birth control methods every couple of years.

1. False
2. True

g15. Which birth control method(s) prevent transmission of HIV? *Pick all that apply.*

1. Depo Provera
2. Pills
3. Ring
4. Patch
5. IUD
6. Implant (Implanon)
7. Condoms
8. Withdrawal
9. Female sterilization (tubal ligation)
10. Male sterilization (vasectomy)
11. Emergency contraception
12. Monogamy

g16. Which of the following birth control methods are the most effective at preventing pregnancy? *Pick three methods.*

1. Depo Provera
2. Pills
3. Ring
4. Patch
5. IUD
6. Implant (Implanon)
7. Condoms
8. Emergency contraception

**IX. HIV Medication Use (Section H)**

**HInst1**. *The next questions will ask about your HIV medications. Telling us about your actual experiences with HIV medications (for example how hard it is) will help us to get a better understanding of what is needed to improve HIV care.*

H1. Have you ever taken HIV medications (also known as antiretroviral medications or ART or HAART)? (If value = No, skip to H9)

1. No
2. Yes

H2. Are you *currently* taking HIV medications? (If value = No, skip to H9)

1. No
2. Yes

H3. How long have you been taking HIV medications?

1. Less than one year
2. 1 to 2 years
3. 2 to 5 years
4. 5 to 10 years
5. Over 10 years

H4. Has your overall health status gotten better or worse since beginning HIV medications?

1. Gotten better
2. Gotten worse
3. No change

H5. Since being on HIV medications, how often do you use condoms?

1. More often than before I was on HIV medications
2. Same
3. Less often
4. I was HIV positive before I began having sex

H6. Since being on HIV medications, how often do you tell your sexual partners about your HIV status?

1. More often than before I was on HIV medications
2. Same
3. Less often
4. I was HIV positive before I began having sex

H7. Since being on HIV medications, has your desire to get pregnant changed?

1. More desire to get pregnant
2. Same
3. Less desire
4. I was HIV positive before I began having sex

H8. Since being on HIV medications, has your sexual desire changed?

1. Greater sexual desire
2. Same
3. Less sexual desire
4. I was HIV positive before I began having sex

H9. Has your doctor or nurse practitioner ever recommended that you start taking HIV medications and you decided that it was not the right time for you?

1. No
2. Yes

H10. Approximately what is the risk of giving HIV to your partner if you’re on HIV medications (during sex without a condom)?

1. 100% (My partner will always/definitely get HIV)
2. 75%
3. 50%
4. 25%
5. 10%
6. 5%
7. 2%
8. No chance

H11. Approximately what is the risk of giving HIV to your partner if you’re *NOT* on HIV medications (during sex without a condom)?

1. 100% (My partner will always/definitely get HIV)
2. 75%
3. 50%
4. 25%
5. 10%
6. 5%
7. 2%
8. No chance

H12. Approximately what is the risk of giving HIV to your baby if you’re on HIV medications (during pregnancy)?

1. 100% (My baby will definitely get HIV)
2. 75%
3. 50%
4. 25%
5. 10%
6. 5%
7. 2%
8. No chance

H13. Approximately what is the risk of giving HIV to your baby if you’re *NOT* on HIV medications (during pregnancy)?

1. 100% (My baby will definitely get HIV)
2. 75%
3. 50%
4. 25%
5. 10%
6. 5%
7. 2%
8. No chance

**XI. Communication (Section J)**

**jInst1**. *Thank you for your honesty so far. The next series of questions is about communication.*

J1. In the past 6 months, did you talk with any *friends and* *family members* about any of these subjects? *Check all that apply.*

1. Condom use
2. Birth control
3. std testing
4. HIV testing
5. None of these

**JInst2**. *For the next twelve questions, please indicate whom you have told about your HIV status. If you do not have a particular relationship, please select ‘Does not apply’.*

|  | **Disclosed HIV status to**: | Told **none** of them (1) | Told **some** of them (2) | Told **all** of them (3) | Does not apply (4) |
| --- | --- | --- | --- | --- | --- |
| J2. | Parent(s) | 🞏 | 🞏 | 🞏 | 🞏 |
| J3. | Brothers or sisters(s) | 🞏 | 🞏 | 🞏 | 🞏 |
| J4. | Primary or steady sexual partner(s) | 🞏 | 🞏 | 🞏 | 🞏 |
| J5. | Other sexual partner(s) | 🞏 | 🞏 | 🞏 | 🞏 |
| J6. | Children | 🞏 | 🞏 | 🞏 | 🞏 |
| J7. | Extended family (e.g., cousins, Aunts and uncles) | 🞏 | 🞏 | 🞏 | 🞏 |

| J8. | Closest friend(s) | 🞏 | 🞏 | 🞏 | 🞏 |
| --- | --- | --- | --- | --- | --- |
| J9. | Other friends | 🞏 | 🞏 | 🞏 | 🞏 |
| J10. | People at work | 🞏 | 🞏 | 🞏 | 🞏 |
| J11. | Landlord | 🞏 | 🞏 | 🞏 | 🞏 |
| J12. | Health care provider(s) | 🞏 | 🞏 | 🞏 | 🞏 |
| J13. | Neighbors | 🞏 | 🞏 | 🞏 | 🞏 |

J14. Have you ever had unprotected sex without telling your partner about your HIV status?

1. No
2. Yes

J15. Have you ever had unprotected sex with your partner even though he/she was aware of your HIV status?

1. No
2. Yes

J16. Have you ever transmitted HIV to a sexual partner (that you know of)?

1. No
2. Yes

J17. How sure/confident are you could ask your partner to get tested for HIV?

1. Very sure
2. sure
3. Not sure
4. Very unsure

J18. How sure/confident are you that you could ask your partner to use a condom?20

1. Very sure
2. sure
3. Not sure
4. Very unsure

J19. How sure/confident are you that you could ask your partner to use a condom if he does not want to use one?21

1. Very sure
2. sure
3. Not sure
4. Very unsure

J20. Is there a difference in how easy it is to discuss condom use with a long-term partner versus a casual partner?22

1. It is easier to discuss with a casual partner
2. It is easier to discuss with someone I am in a committed relationship with
3. There is no difference

J21. If you were given the choice, how would you want your partner to be told that he should get tested and treated for HIV? 23

1. Tell him myself
2. Have a provider (doctor, nurse, health educator) tell him
3. I wouldn’t want him to be told

J22. I think it is my responsibility to tell my partner about my HIV status. 24

1. strongly agree
2. Agree
3. Disagree
4. strongly disagree

J23. If you were given a referral sheet for your partner telling him where he could go for HIV testing and treatment, would you give it to him?25

1. Yes
2. No

J24. Would you be comfortable hearing and talking about birth control, sex, and STD prevention in a group setting?26

1. No
2. Yes

J25. I think it is important for my partner to know if I get an STD.

1. strongly agree
2. Agree
3. Disagree
4. strongly disagree

J26. Do you think your partner would tell you if he had an STD?

1. Yes
2. No

J27. In your relationships, who usually makes (or made) decisions about whether to get pregnant?

1. I do (did)
2. He does (did)
3. we both do (did) equally

J28. In your relationships, who usually makes (or made) decisions about whether to use birth control?

1. I do (did)
2. He does (did)
3. we both do (did) equally

J29. In your relationships, who usually makes (or made) decisions about using condoms?

1. I do (did)
2. He does (did)
3. we both do (did) equally

J29. In your relationships, who usually makes (or made) decisions about using condoms?

1. I do (did)
2. He does (did)
3. we both do (did) equally

J29. In your relationships, who usually makes (or made) decisions about using condoms?

1. I do (did)
2. He does (did)
3. we both do (did) equally

J30. During the past month, have you often been bothered by feeling down, depressed, or hopeless?

1. No
2. Yes

J31. During the past month, have you often been bothered by little interest or pleasure in doing thing?

1. No
2. Yes

**JInst3.** You’re all done with the survey! Thank you very much for your participation! Please let the research staff member know you have completed the survey.

**XIII. Data Entry (Z Section)**

**Zinst1.** *Directions to Research Assistants: Please refer to the individual codes assigned to your site when completing the data entry screen. Please also complete the participant tracking sheet for each ACASI administration.*

Z1. Enter the Participants ID Number ####

Z2. Enter the Date: MM/DD/YYYY

Z3. Enter the laptop ID number ###

Z4. Enter Research Assistant Code - enter initials (ex: DU)

Z5. Enter the survey completion time. _________ Minutes
